# Food insecurity and coping strategies associate with higher risk of anxiety and depression among South African households with children

**DOI:** 10.1017/S1368980024000879

**Published:** 2024-04-05

**Authors:** Siphiwe N Dlamini, Asanda Mtintsilana, Ashleigh Craig, Witness Mapanga, Shane A Norris

**Affiliations:** 1 School of Physiology, Faculty of Health Sciences, University of the Witwatersrand, Johannesburg, South Africa; 2 SAMRC/Wits Developmental Pathways for Health Research Unit, Faculty of Health Sciences, University of the Witwatersrand, Johannesburg, South Africa; 3 Strengthening Oncology Services Research Unit, Faculty of Health Sciences, University of the Witwatersrand, Johannesburg, South Africa; 4 School of Human Development and Health, University of Southampton, Southampton, UK

**Keywords:** Food insecurity, Coping strategies, Households with children, Anxiety, Depression

## Abstract

**Objective:**

To investigate food insecurity and related coping strategies, and their associations with the risk of anxiety and depression, among South African households with children.

**Design:**

Nationally representative cross-sectional study. Tools for assessing food insecurity, coping strategies, risk of anxiety and depression were assessed from the Community Childhood Hunger Identification Project, Coping Strategies Index, Generalised Anxiety Disorder-7 and Patient Health Questionnaire-9, respectively. We used ordered logistic regression to test associations of food insecurity and coping strategies with the risk of anxiety and depression. Moderating effects of each coping strategy were tested in the associations of food insecurity with anxiety and depression.

**Setting:**

South Africa, post COVID-19 restrictions, May–June 2022.

**Participants:**

1,774 adults, weighted to 20,955,234 households.

**Results:**

Food insecurity prevalence was 23·7 % among households with children. All coping strategies were used to some extent, but relying on less preferred and less expensive foods was the most used strategy (85·5 % of food-insecure households). Moving to a higher level of food insecurity was associated with >1·6 greater odds of being in a higher risk of anxiety and depression. Sending a household member to beg for food was the strongest associated factor (OR = 1·7, *P* < 0·001). All coping strategies partly moderated (lessened) the associations of food insecurity with a higher risk of anxiety and depression.

**Conclusions:**

Food insecurity among households with children was high following the COVID-19 pandemic. Collaborative efforts between government, private sector and civil society to eradicate food insecurity should prioritise poorer households with children, as these populations are the most vulnerable.

Food insecurity – the state of not having access to sufficient food due to limited money or other resources – is a growing global health concern following recent global events of a pandemic and global economic crises^(1)^. Low-resource settings like those of sub-Saharan African countries remain disproportionally affected^(1,2)^. Living in a food-insecure household is associated with adverse long-term health consequences like a higher risk of obesity and cardiometabolic diseases^(3)^ but also more immediate health threats like a higher risk of impaired mental health including anxiety and depression^(4)^. The impact of food insecurity on these adverse health outcomes may be partly related to the strategies used by household members to deal with food access issues^(5)^. For example, coping strategies that often start earlier, and are used to prevent household food insecurity, include relying on cheaper foods and/or skipping meals, which can directly influence nutrition quality^(6,7)^. Likewise, having to beg for food can directly impact one’s mental health^(5)^. Food insecurity among households with children is even more concerning because when a child has inadequate access to food, nutritional status is greatly affected, leading to a negative impact on the child’s physical, emotional and cognitive development^(8–10)^.

South Africa (SA) has the greatest inequality in the world^(11)^. We recently used a nationally representative study to assess the state of food insecurity and associated coping strategies in SA, during the shift out of the pandemic with low-level COVID-19 lockdown restrictions in October 2021^(5)^. We found that 20·4 % of South African households were food insecure, and the prevalence was largely dependent on socio-economic factors^(5)^. However, whether the high rates of food insecurity were attributed to lockdown restrictions was not investigated. It is also possible that households with children were disproportionally affected by food insecurity, as suggested by previous global reports^(1)^. Our previous study also demonstrated that living in a food-insecure household was associated with an increased risk of anxiety and depression^(5)^. Additionally, all coping strategies were found to associate with a higher risk of anxiety and depression, and sending a household member to beg for food was the strongest predictor^(5)^. On the 4th of April 2022, the South African Government announced the end of COVID-19 lockdown restrictions^(12)^. To the best of our knowledge, there are no nationally representative studies in SA that have investigated the impact of food insecurity and related coping strategies on mental health among households with children, post COVID-19 lockdown restrictions and the start of the global economic crisis.

Hence, the primary aim of this study was to investigate the state of food insecurity and related coping strategies among South African households with children, after the COVID-19 lockdown restrictions had ended and the global economic crises precipitated by high energy costs and the Russian–Ukrainian war had begun^(13)^. While it is likely that the associations between food insecurity and a higher risk of impaired mental health were moderated by the associated coping strategies^(5)^, there are no studies that have investigated the influence of the commonly used coping strategies on the relationships between food insecurity and the risk of anxiety and depression. Therefore, the study also aimed to assess whether the relationships between food insecurity and the risk of anxiety and depression were moderated by the related coping strategies.

## Methods

### Study design and setting

This was a cross-sectional study of a nationally representative survey, comprising of a total of 3 459 adult (aged 18 years and above) respondents, who were interviewed between May and June 2022. However, for the present analyses, only respondents who indicated that they were living with children (at least one ≤17 years old) were included (*n* 1 774). A six-phase stratified random probability sampling approach was used, with the details described elsewhere^(14)^ and summarised in online supplementary material, Supplemental Fig. S1. Face-to-face interviews were conducted via computer-assisted personal interviewing technology, across all nine South African provinces. The interviewers were trained to collect research data by IPSOS (www.ipsos.com), an international research company. The interviewers were able to conduct the interviews in the respondent’s preferred local languages.

### Survey questionnaire

The questionnaire included sections about households (province and community type, food insecurity and related coping strategies), as well as items that were only related to individual respondents (age, gender, level of education, employment status and self-reported ethnicity). Sections about food insecurity, related coping strategies and levels of anxiety and depression were all adapted from standard and validated questionnaires. We used the Community Childhood Hunger Identification Project (CCHIP) tool^(15,16)^ to access food insecurity and the Coping Strategies Index tool^(17)^ to assess coping strategies. Briefly, the Coping Strategy Index tool is a standard questionnaire for assessing how household cope against periods of limited food access. The tool comprises eleven individual items, and the respondents are required to indicate how frequently they use each of the coping strategy. The frequency options include ‘Less than once a week’, ‘1–2 times per week’, ‘3–6 times per week’, ‘Everyday’ and ‘Never’. To screen for symptoms of anxiety and depression, we used the Generalised Anxiety Disorder (GAD-7)^(18)^ and the Patient Health Questionnaire-9 (PHQ-9)^(19)^, respectively.

### Food insecurity, anxiety and depression groups

#### Food insecurity groups

The CCHIP tool comprised questions relating to food insecurity, experienced in the previous 12 months^(15)^. A food insecurity score was computed using the following four CCHIP questions, with a point of one assigned for each ‘Yes’ response: (1) Does your household ever run out of money to buy food?; (2) Do you ever cut the size of meals or skip meals because there is not enough money for food?; (3) Do your children ever say they are hungry because there is not enough food in the house? and (4) Do you or any of your children ever go to bed hungry because there is not enough money to buy food? Subsequently, households with a food insecurity score of zero (those who responded with a ‘No’ to all four CCHIP questions) were classified as ‘Food Secure’. In contrast, households with a score of 1 or 2 were classified as ‘At Risk’, and those with a score of 3 or 4 were classified as ‘Food Insecure’.

#### Risk of anxiety and depression groups

We used the GAD-7 scores to categorise the respondents into four risk of anxiety groups as follows: 0–4 = minimal anxiety, 5–9 = mild anxiety, 10–14 = moderate anxiety and 15–21 = severe anxiety^(18)^. Likewise, we used the PHQ-9 scores to categorise the respondents into five risk of depression groups as follows: 0–4 = minimal depression, 5–9 = mild depression, 10–14 = moderate depression, 15–19 = moderately severe depression and 20–27 = severe depression^(19)^.

### Statistical analysis

All statistical analyses were conducted in STATA 17.0 (StataCorp). Using a random interactive method^(20)^, data were weighted to represent the most recent census of the South African adult population (18 years and older, *n* 20 955 234)^(21)^. Province, home language, ethnicity, gender and age were all included as variables of the weighing matrix^(5)^. Ordered logistic regression models were used to test associations between predictors and outcomes. In the first set of models, we tested the associations of food insecurity with risk of anxiety and depression, where the food insecurity group (food secure = 0, at risk = 1 and food insecure = 2) was the predictor, and the levels of anxiety (minimal = 0, mild = 1, moderate = 2 and severe = 3) and depression (minimal = 0, mild = 1, moderate = 2, moderately severe = 3 and severe = 4) were the individual outcomes. In the second set of models, we tested the associations of food insecurity with risk of anxiety and depression while adjusting for each coping strategy, to test the coping strategy’s moderating effects. In the third set of models, we tested the associations of each coping strategy with risk of anxiety and depression, where each coping strategy score was included as a predictor, and the levels of anxiety and depression were included as individual outcomes. A two-tailed test was considered statistically significant when *P* was <0·050.

## Results

### Basic characteristics of the study sample

Basic characteristics of the study sample are shown in Table 1. Using weighted data, the study sample was largely comprised of female respondents (60·6 %), with a median age of 35 years. Most of the respondents (80·1 %) were self-identified as black, with the smallest ethnic group being Asian (2·9 %). Only about half of the respondents had completed Grade 12/Matric (53·3 %) and also half were employed (46·5 %). The majority of the interviewed respondents (48·2 %) resided within metropolitan areas (municipality areas with all the operational functions of a local government). Regarding food insecurity, only about half of the respondents were classified as being food secure (53·1 %). Regarding mental health, only 56·8 % and 50·0 % of the respondents were classified as having minimal anxiety and depression, respectively.

**Table 1.** Basic characteristics of the study sample

	Unweighted (*n* 1 774)		Weighted (20 955 234)	
	*n*/median	%/IQR	*n*/median	%/IQR
Gender				
* Male*	710	40·0	8 258 474	39·4
* Female*	1 064	60·0	12 696 760	60·6
Age (years)	35	28–44	35	36–45
Ethnicity				
* White*	118	6·7	1 653 557	7·9
* Black*	1 420	80·0	16 791 606	80·1
* Asian*	38	2·1	600 644	2·9
* Coloured*	198	11·2	1 909 427	9·1
Education				
* No schooling*	14	0·8	191 075	0·9
* Some primary school*	21	1·2	433 449	2·1
* Primary school completed*	34	1·9	484 828	2·3
* Some high school*	404	22·8	5 042 625	24·1
* Matric/grade 12*	974	54·9	11 174 740	53·3
* Technikon diploma/degree completed*	166	9·4	1 893 302	9·0
* University degree completed*	113	6·4	1 272 643	6·1
* Other post-matric qualification*	43	2·4	401 091	1·9
* NSC/short course only*	5	0·3	61 481	0·3
Employment status				
* Unemployed*	723	40·8	8 625 042	41·1
* Retired*	68	3·8	1 102 728	5·3
* Employed*	879	49·5	9 751 197	46·5
* Student*	104	5·9	1 476 267	7·0
Community type				
* Metropolitan*	1 035	58·3	10 100 734	48·2
* Urban*	255	14·4	3 851 351	18·4
* Rural*	484	27·3	7 003 149	33·4
Food security group				
* Food secure*	958	54·0	11 136 475	53·1
* At risk*	420	23·7	4 857 661	23·2
* Food insecure*	396	22·3	4 961 098	23·7
Anxiety categories				
* Minimal*	1 012	57·0	11 895 187	56·8
* Mild*	483	27·2	5 720 653	27·3
* Moderate*	220	12·4	2 639 367	12·6
* Severe*	59	3·3	700 027	3·3
Depression categories				
* Minimal*	895	50·5	10 473 624	50·0
* Mild*	438	24·7	5 261 361	25·1
* Moderate*	268	15·1	3 325 550	15·9
* Moderately severe*	132	7·4	1 441 574	6·9
* Severe*	41	2·3	453 125	2·2

Data were weighted to represent the most recent census of the South African adult population (18 years and older, *n* 20 955 234). Province, home language, ethnicity, gender and age were all included as variables of the weighing matrix. IQR: Interquartile range.

### Food insecurity among South African households with children

**Fig. 1:**
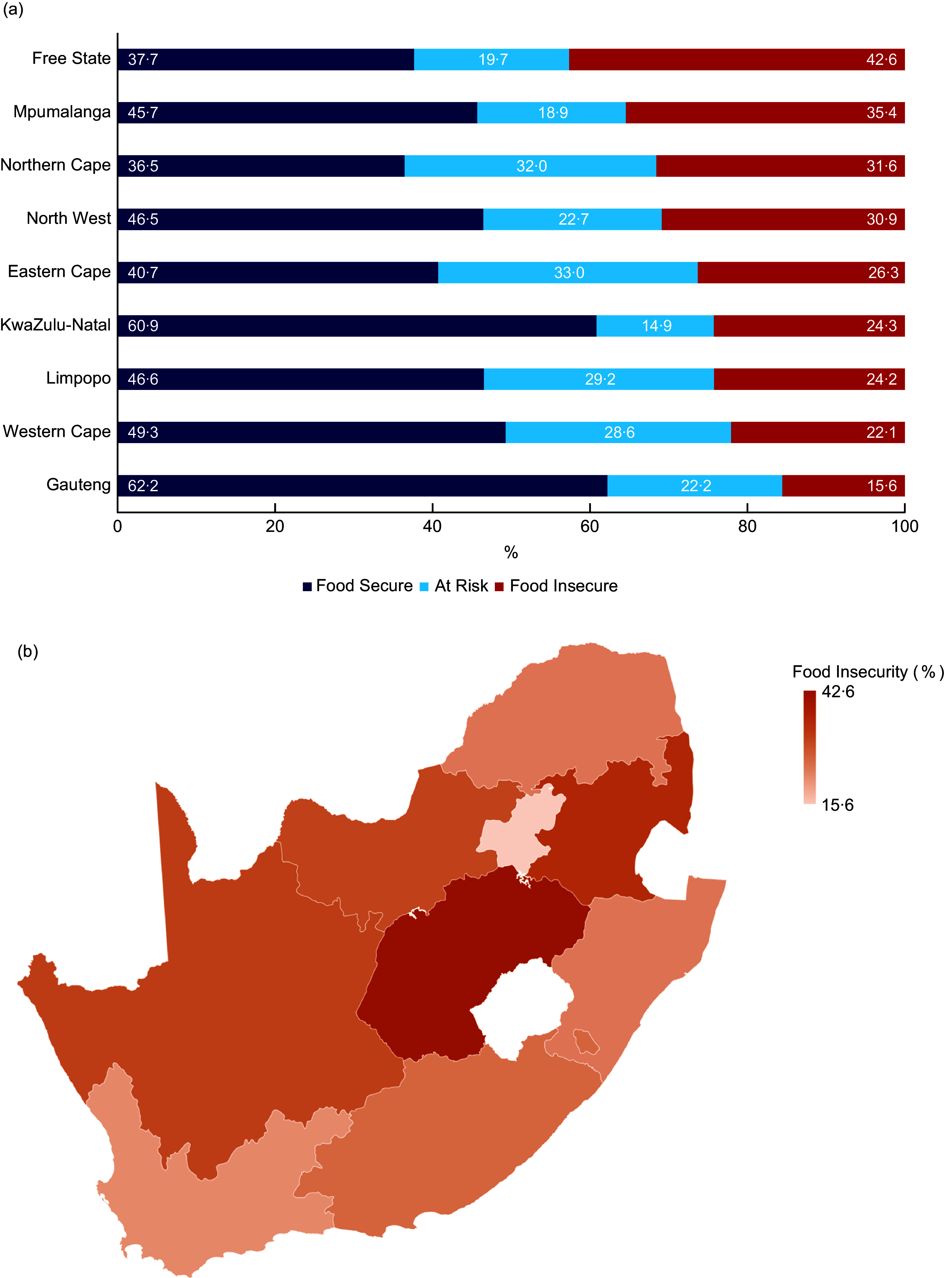
Prevalence of food insecurity across all South African provinces among households with children

Table 2 shows that a large proportion of South African household with children experienced food insecurity-related issues. For example, most of the households (about 40·4 %) indicated that they often ran out of money to buy food. About 23·7 % of South African households with children were classified as being food insecure (Table 1). Correspondingly, about 23·2 % were at risk, and only 53·1 % were food secure (Table 1). Figure 1 summarises food insecurity categories among all South African provinces. Free State was identified as the province with the highest rate of food insecurity (42·6 %), with Gauteng showing the lowest rate (15·6 %).

**Table 2.** Summary of responses to the CCHIP questions among South African households with children

	Yes (%)	If yes, happened in the past 30 d? (%)	If yes, has it happened 5 or more days in the past 30 d? (%)
Does your household ever run out of money to buy food?	40·4	74·6	61·7
Do you ever cut the size of meals or skip meals because there is not enough money for food?	34·5	76·5	72·8
Do your children ever say they are hungry because there is not enough food in the house?	27·2	68·5	85·6
Do you or any of your children ever go to bed hungry because there is not enough money to buy food?	17·8	71·8	91·4

Questions were taken from the CCHIP: Community Childhood Hunger Identification Project questionnaire^(15,16)^.

### Food insecurity by socio-demographics

**Fig. 2:**
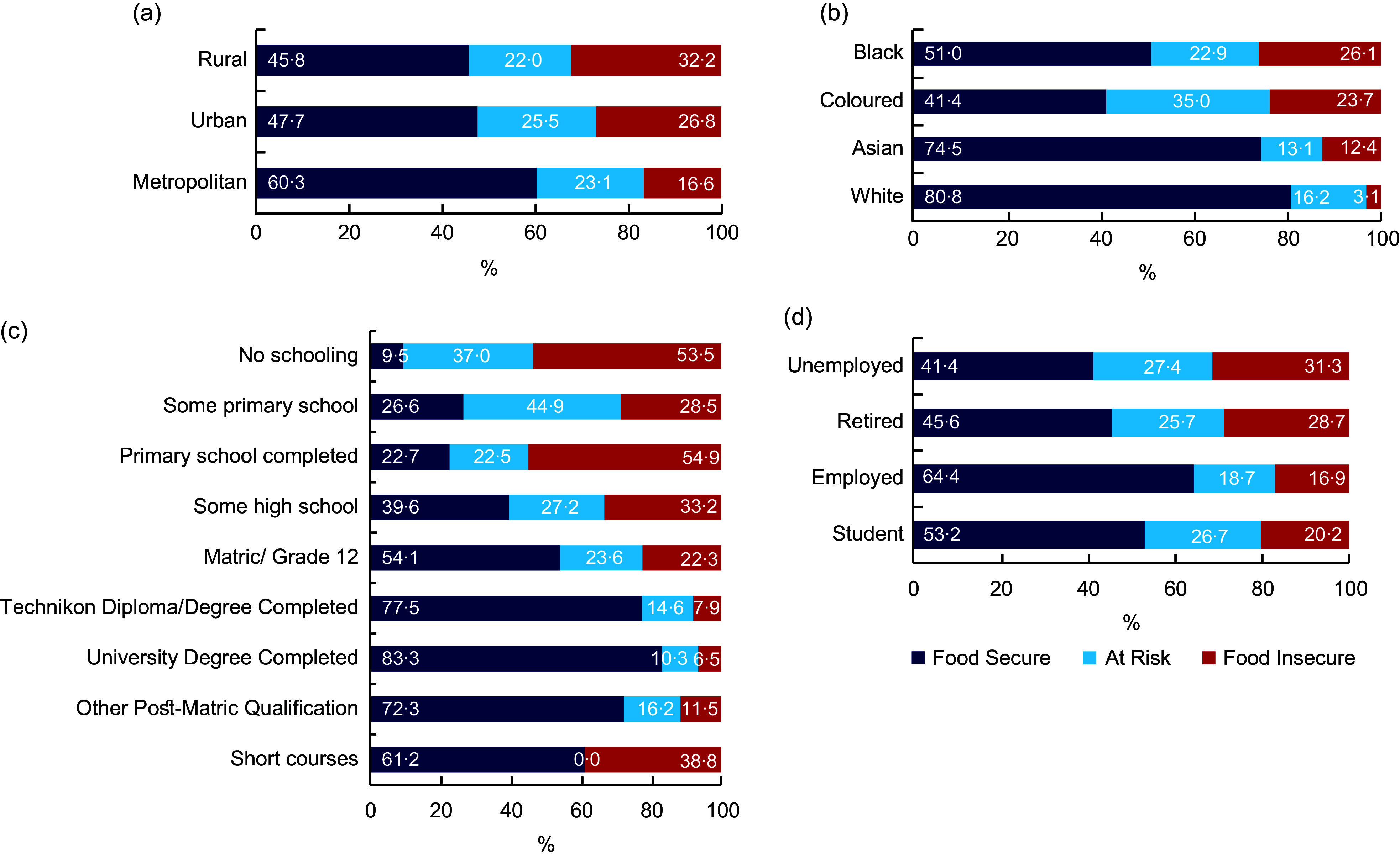
Prevalence of food insecurity by community type (a), ethnicity (b), level of education (c) and employment status (d) among South African households with children

Figure 2 compares food insecurity groups by community type, ethnicity, level of education and employment status. The prevalence of food insecurity was highest in the rural areas (32·2 %), and among respondents who had no formal schooling (53·5 %) and those who were unemployed (31·3 %). Regarding ethnic differences, the Black population had the highest rate of food insecurity (26·1 %). In contrast, the lowest rate of food insecurity was observed in the White population (only 3·1 %).

### Food-related coping strategies

**Fig. 3:**
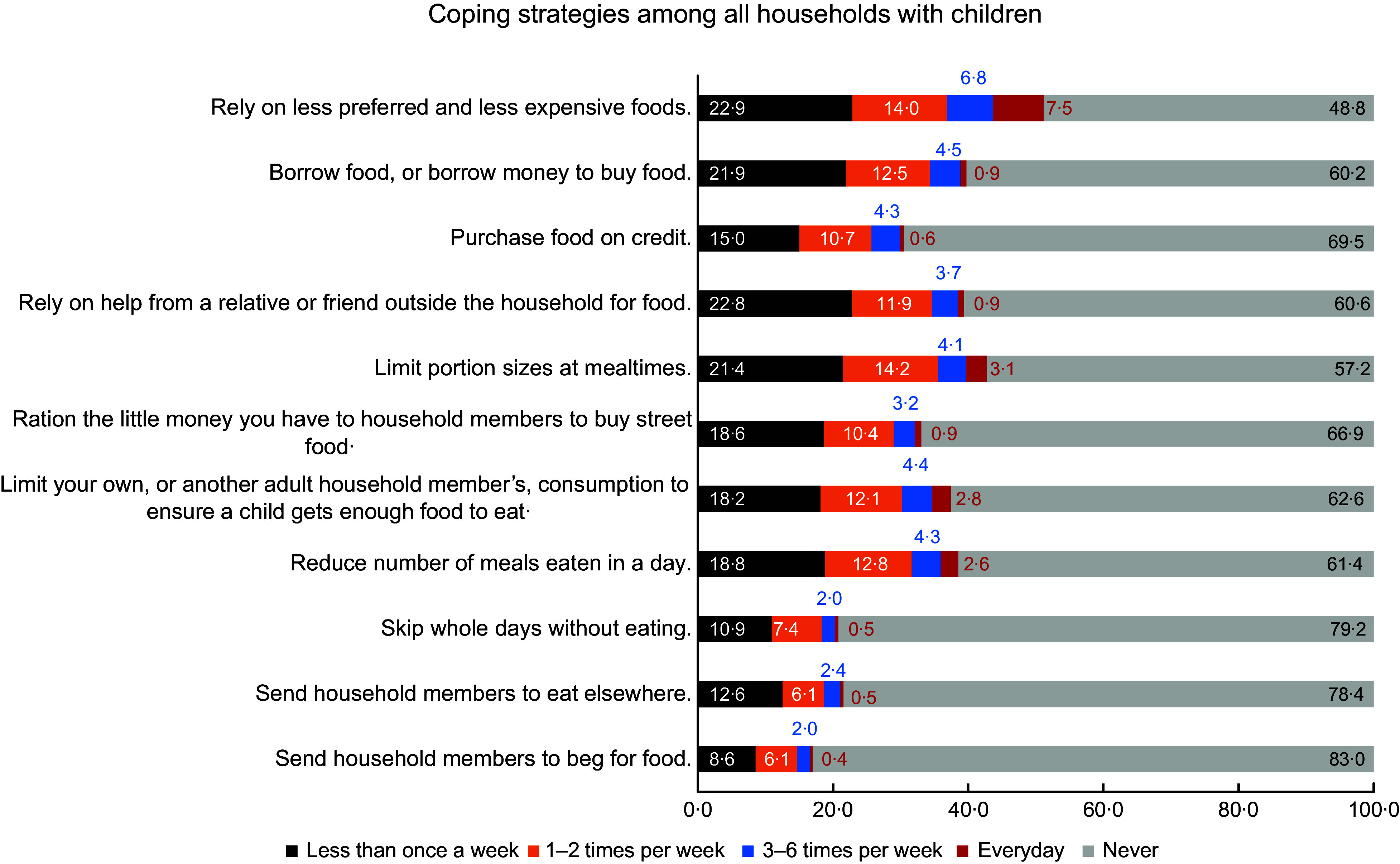
Common coping strategies used by all South African households with children

**Fig. 4:**
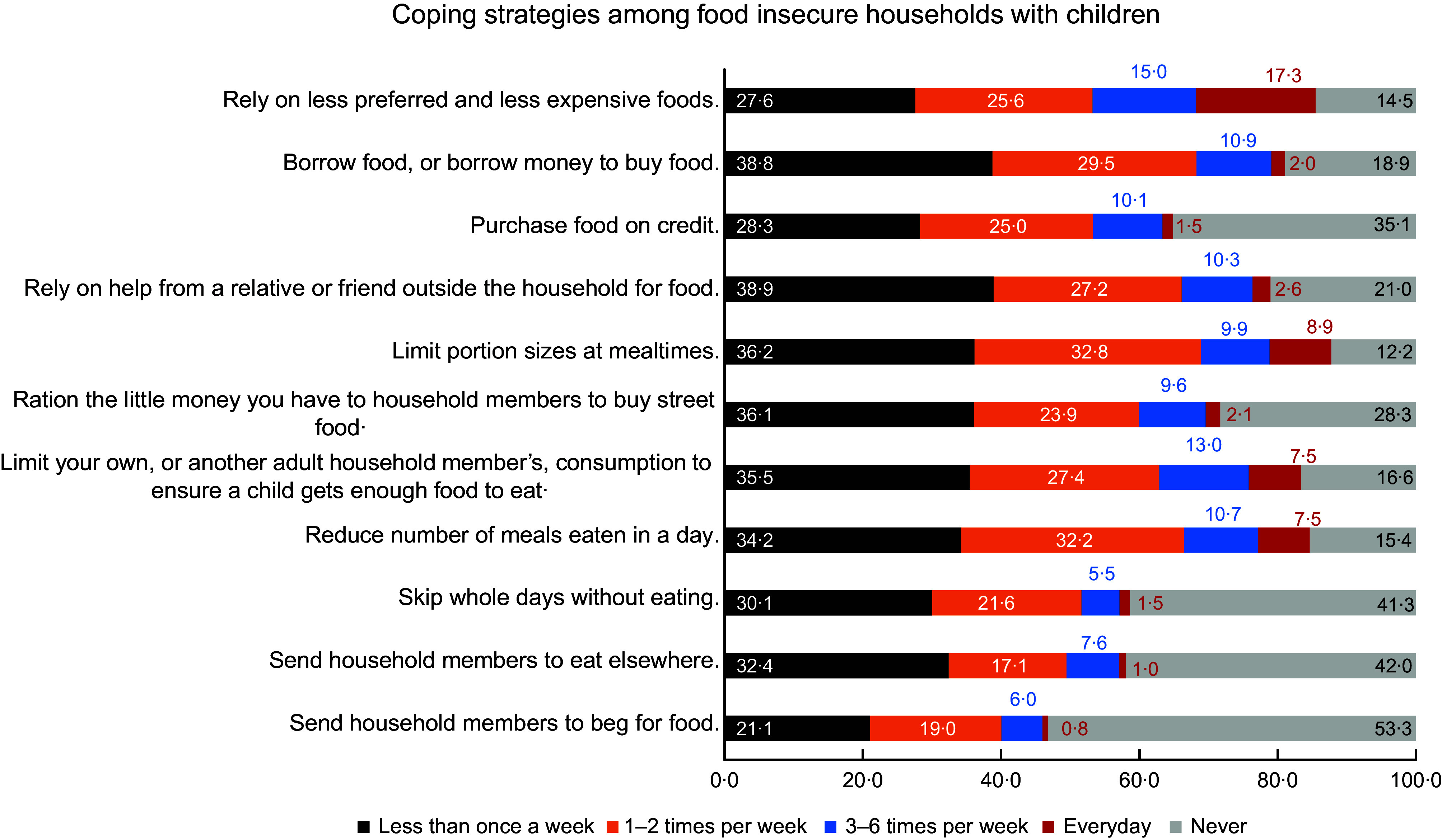
Coping strategies among food-insecure South African households with children

Common coping strategies that are used by all South African households with children are summarised in Fig. 3. Among all included coping strategies, the most common was ‘relying on less preferred and less expensive foods’, which was used by 51·2 % of the households. In contrast, the least used coping strategy was ‘sending a household member to beg for food’, which was only used by 17·0 % of the respondents. Figure 4 shows that all included coping strategies were much more common among the food-insecure households, with 85·5 % relying on less preferred and less expensive foods, and 46·7 % sending a household member to beg for food. We also found that 67·0 % of all households with children used at least one of the included coping strategies, and 50·6 % of all households had to use three or more strategies (results not shown).

### Associations of food insecurity and coping strategies with risk of anxiety and depression

Table 3 summarises associations of food insecurity with levels of anxiety and depression. One level increase in food insecurity category (moving from being ‘food secure’ to being ‘at risk’, or from being ‘at risk’ to being ‘food insecure’) was associated with 1·7 times greater odds of being in a higher level of experiencing anxiety, and 1·6 times greater odds of being in a higher level of experiencing depression. The odds ratio (OR) of food insecurity for risk of anxiety and depression after adjusting for each coping strategy are also shown in Table 3. All coping strategies had some moderating effects (shown by reducing the OR) on the associations between food insecurity and moving to a higher level of anxiety and depression. For example, ‘limiting portion sizes’ was found to have the greatest moderating effects (OR reduced from 1·7 to 1·4) on the association between food insecurity and anxiety. Regarding depression, ‘borrowing food or money to buy food’ had the greatest moderating effects (OR reduced from 1·7 to 1·4).

**Table 3.** Associations of food insecurity with levels of anxiety and depression, and moderating effects of coping strategies

Coping strategy (potential moderator)	Anxiety (outcome)		Depression (outcome)	
	OR	95 % CI	OR	95 % CI
No moderator	1·712	1·711, 1·714	1·633	1·631, 1·634
With moderators				
Rely on less preferred and less expensive foods?	1·450	1·495, 1·498	1·404	1·403, 1·406
Borrow food, or borrow money to buy food?	1·449	1·447, 1·451	1·350	1·349, 1·352
Purchase food on credit?	1·540	1·538, 1·542	1·479	1·477, 1·481
Rely on help from a relative or friend outside the household for food?	1·412	1·411, 1·414	1·399	1·397, 1·400
Limit portion sizes at mealtimes?	1·411	1·410, 1·413	1·359	1·357, 1·360
Ration the little money you have to household members to buy street food?	1·470	1·468, 1·472	1·408	1·406, 1·409
Limit your own, or another adult household member’s, consumption to ensure a child gets enough food to eat?	1·438	1·436, 1·440	1·356	1·354, 1·358
Reduce number of meals eaten in a day?	1·426	1·425, 1·428	1·367	1·365, 1·368
Skip whole days without eating?	1·564	1·563, 1·566	1·470	1·468, 1·472
Send household members to eat elsewhere?	1·543	1·541, 1·544	1·420	1·419, 1·422
Send household members to beg for food?	1·507	1·506, 1·509	1·427	1·425, 1·428

Ordered logistic regression was used with food insecurity group as the predictor and Generalised Anxiety Disorder-7 (anxiety) and Patient Health Questionnaire-9 (depression) categories as the outcomes. All *P* values for the OR were <0.001. CI: Confidence Interval.

Table 4 summarises the associations of each coping strategy with levels of anxiety and depression. Although the use of each coping strategy was associated with greater risk of both anxiety and depression, ‘sending a household member to beg for food’ was associated with the greatest odds of being in a higher level of anxiety and depression (both OR were about 1·7). In contrast, the strategy that was associated with lowest odds was ‘relying on less preferred and less expensive foods’ (both OR were about 1·4). We also found that the prevalence of probable anxiety (GAD-7 score ≥10) and probable anxiety (PHQ-9 score ≥10) was higher among households who used three or more coping strategies, compared with those who used less (22·6 % *v*. 9·1 % for probable anxiety and 33·7 % *v*. 15·9 % for probable depression). These were results that are not shown in figures and tables.

**Table 4.** Associations of coping strategies with levels of anxiety and depression

Coping strategy (predictor)	Anxiety (outcome)		Depression (outcome)	
	OR	95 % CI	OR	95 % CI
Rely on less preferred and less expensive foods?	1·365	1·364, 1·365	1·376	1·375, 1·377
Borrow food, or borrow money to buy food?	1·573	1·572, 1·575	1·588	1·587, 1·590
Purchase food on credit?	1·480	1·479, 1·481	1·443	1·441, 1·444
Rely on help from a relative or friend outside the household for food?	1·670	1·665, 1·669	1·542	1·541, 1·544
Limit portion sizes at mealtimes?	1·522	1·521, 1·523	1·490	1·489, 1·491
Ration the little money you have to household members to buy street food?	1·599	1·597, 1·600	1·559	1·558, 1·561
Limit your own, or another adult household member’s, consumption to ensure a child gets enough food to eat?	1·509	1·508, 1·511	1·504	1·503, 1·505
Reduce number of meals eaten in a day?	1·521	1·520, 1·523	1·489	1·487, 1·490
Skip whole days without eating?	1·543	1·541, 1·544	1·556	1·554, 1·557
Send household members to eat elsewhere?	1·581	1·579, 1·582	1·678	1·676, 1·680
Send household members to beg for food?	1·734	1·732, 1·736	1·737	1·735, 1·739

Ordered logistic regression was used with each coping strategy as the predictor and Generalised Anxiety Disorder-7 (anxiety) and Patient Health Questionnaire-9 (depression) categories as the outcomes. All *P* values for the OR were <0.001.

## Discussion

In the present study, we investigated the state of food insecurity and related coping strategies, and their associations with risk of anxiety and depression, among South African households with children, after COVID-19 lockdown restrictions had ended. We found that food insecurity among South African households with children was more than 1 in 5 (about 23·7 %). Also, we demonstrated that being food insecure was associated with higher risk of anxiety and depression, even after the lockdown restrictions had ended. To the best of our knowledge, this is the first study to show that the relationships of food insecurity with higher risk of anxiety and depression were partly moderated by coping strategies. Using each coping strategy was associated with a higher risk of impaired mental health, such that about 22·6 % and 33·7 % of respondents from households that used three or more coping strategies had probable anxiety and depression, respectively.

Our observation that 23·7 % (more than 1 in 5) of South African households with children were food insecure is in accordance with recent national findings. In a nationally representative survey, we found that 20·4 % of South African households were food insecure^(5)^. The slightly higher rate of food insecurity in the present study is most likely attributed to the fact that only households with children were included, and households with children are often more affected by food insecurity^(22,23)^. Using the CCHIP tool, a South African study reported that about 32·5 % of South African households with children were food insecure between the years 2011 and 2012^(23)^. Similarly, Statistics SA recently reported that, while about 20·9 % of the South African households were classified as either having inadequate or severely inadequate food access in 2021, the rate increased to 27·1 % when only households with children were included^(22)^.

The higher rates of food insecurity in many sub-Saharan African countries were attributed to the previously implemented COVID-19 restrictions, which led to economic disruptions like disturbances in food supply chains but also loss of livelihoods and income, especially among people in poor resource settings^(24,25)^. Against this background, food access was expected to improve after the restrictions had ended^(26)^. However, the few studies that investigated food-related issues after lockdown restrictions had ended were conducted in high-income countries, and the findings were similar to what is reported in the present study, suggesting that the rate of food insecurity continues to increase^(27,28)^. For example, a repeated cross-sectional study conducted in Australia demonstrated that the rate of food insecurity had increased from 19·5 % when lockdown restrictions were at a low level to 22·6 % when the restrictions had ended^(28)^. To the best of our knowledge, this is the first study, from a sub-Saharan country, to confirm that food insecurity had worsened after the lockdown restrictions had ended. It is unlikely that the state of food insecurity will improve in the near future. This is because the COVID-19 pandemic led to negative changes in key economic areas, like agriculture and food production, that are likely to continue for many years^(29)^.

Regardless of the country’s income status, socio-economic factors play important roles in determining which households are affected by food insecurity^(30,31)^. In the present study, food insecurity was dependent on socio-economic factors, such that respondents who were Black, unemployed and with no formal education were the most food insecure. This has also been shown in our previous nationally representative study conducted during COVID-19 restrictions^(5)^. In these nationally representative studies, assessing socio-economic factors was key because coping strategies used to deal with food insecurity are strongly related to the socio-economic status of household members^(5)^. For example, relying on cheaper foods (the most common coping strategy) is largely dependent on employment status, which is, in turn, influenced by the level of education^(5,32)^. Notably, some of the commonly used coping strategies may greatly influence nutritional intake among the household members. Such strategies include relying on cheaper foods, limiting portion sizes, reducing number of meals and skipping whole days without eating. In accordance with this hypothesis, food insecurity is strongly linked to poor nutritional status among children and adult household members^(33,34)^.

In line with our previous findings^(5)^, we have confirmed that food insecurity associated with a higher risk of anxiety and depression, among South African households with children. The associations between food insecurity and poor mental health outcomes have been reported in many populations, including sub-Saharan African countries like SA^(4,5,35)^. However, the role of coping strategies on these relationships has been less documented. Similar to our previous study that considered all South African households^(5)^, we demonstrated that the most commonly used coping strategy was relying on less expensive and less preferred foods, which was used by more than half (51·2 %) of the households. Accordingly, our observation that the least used strategy was sending a household member to beg for food (used by only 17 %) was also in accordance with our previous report^(5)^. Assessing coping strategies among South African households with children was important in the present study, as each coping strategy may differentially affect the health and development of household children^(36)^. On the one hand, relying on less expensive or less preferred food could compromise the nutritional status of the affected children and have a negative impact on physical development^(37)^. On the other hand, sending a household member to beg for food was the strongest predictor of impaired mental health^(5)^ and could impact the psychological development of the affected children by exposing them to adverse childhood experiences^(38)^.

The relationships between food insecurity, coping strategies and mental health outcomes are complex, as these components are often influenced by several known and unknown factors^(39,40)^. Nevertheless, findings from the present study support the hypothesis that the relationships between food insecurity and impaired mental health are partly moderated by the coping strategies. Although each coping strategy reduced the strength of the relationships to some extent, none of the strategies completely moderated the associations of food insecurity with the mental health outcomes. Hence, it is likely that other factors may play a role in the impact of food insecurity on the risk of anxiety and depression. For example, we have recently demonstrated that the risk of anxiety and depression was strongly associated with adverse childhood experiences in a nationally representative sample of South African adults^(40)^. Likewise, some cultural factors (including career preferences and self-identity) have been shown significantly influence food insecurity in different populations^(41,42)^.

### Study limitations and future directions

The present study has some limitations. This was a cross-sectional study from which causality cannot be inferred in any of the reported associations. To the best of our knowledge, there are no reported studies that compared South African households with and without children after the lockdown restrictions had ended. Therefore, future studies should explore the relationships of food insecurity, coping strategies and impaired mental health between households with and without children, after the lockdown restrictions had ended. The key strength of the study was the use of a nationally representative sample which was weighted to project the entire population of South African households with children.

### Conclusions, implications and recommendation

High rates of food insecurity among households with children are a concern in SA. Collaborative efforts between government, non-government organisations and civil society to eradicate food insecurity should prioritise poorer households with children, as these populations are the most vulnerable. Solutions should include rethinking child support grants, so that they offer more realistic support to households with children. According to the November 2023 Household Affordability Index report, it costs an average of R5 300 to feed one South African family, and this is far more than what is offered by government grants^(43)^. While school feeding schemes, like the Government’s National School Nutrition Programme^(44)^, may reduce food insecurity when poorer students are at school, solutions to improve food access at home are still required. Such solutions could include introducing permanent food aid programmes for all households with children, similar those that were used during the lockdown restrictions^(45)^. In developed nations like the USA, food stamp programmes, pop-up kitchens and discount vouchers have been shown to be promising solutions for improving food access among poorer households^(46–48)^. These programmes can also be used as immediate solutions to reduce food insecurity among poorer South African households with children.

## Supporting information

Dlamini et al. supplementary material Dlamini et al. supplementary material

## Supplementary material

For supplementary material accompanying this paper visit https://doi.org/10.1017/S1368980024000879
