# Supplementary material for: Food insecurity and coping strategies associate with higher risk of anxiety and depression among South African households with children
Source: Public Health Nutr. 2024 Apr 5;27(1):e116. doi: 10.1017/S1368980024000879 (PMC11036448; doi:10.1017/S1368980024000879)
Supplement: Dlamini et al. supplementary material [file S1368980024000879sup001.pdf]

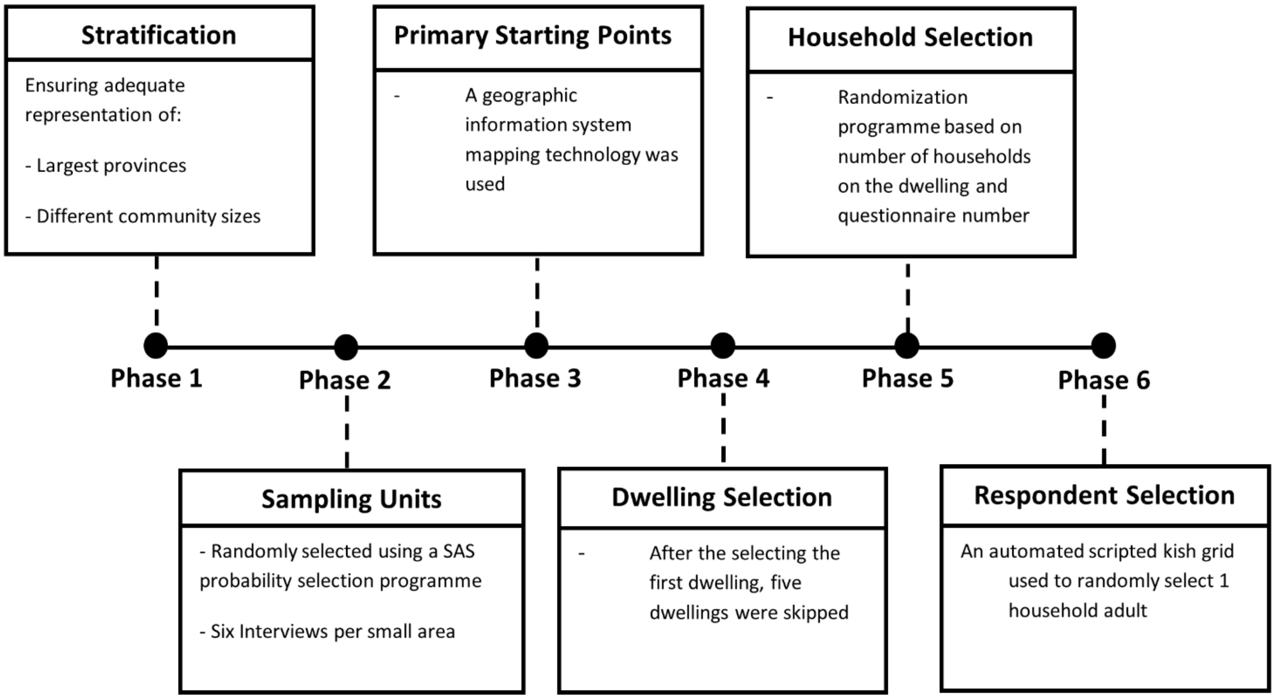

Figure S1: Summary of the sampling method.

Figure adapted from Dlamini et al (5). The study employed a six-phase stratified random probability sampling approach. In Phase 1, stratification was three-staged to ensure adequate representation of the largest provinces, different community sizes, and gender. Phase 2 involved selecting sampling units, defined as small areas merging smaller Enumerated Areas with populations over 500. A SAS probability selection program was used during this phase to randomly select units based on size proportion. Six interviews were conducted per small area. In Phase 3, a geographic information system mapping technology was employed to randomly select starting points (e.g., schools, churches, or prominent buildings) for interviewers to begin their random walks. Phase 4 focused on dwelling selection, with interviewers moving up roads from the starting point based on dwelling numbers. After the first dwelling was selected, five were skipped, and the sixth was selected for an interview. Phase 5 involved household selection, defining a household as a unit with one person living alone or a group living together for at least four nights a week. The randomization program selected households based on dwelling size and questionnaire number. Finally, in Phase 6, respondent selection occurred by listing all household members on a scripted kish grid, excluding those under 18. The automated kish grid then identified the respondent for the interview.
